# Presentation of HIV-1 envelope glycoprotein trimers on diverse nanoparticle platforms

**DOI:** 10.1097/COH.0000000000000549

**Published:** 2019-02-25

**Authors:** Philip J.M. Brouwer, Rogier W. Sanders

**Affiliations:** a Department of Medical Microbiology, Amsterdam UMC, University of Amsterdam, Amsterdam, the Netherlands; b Department of Microbiology and Immunology, Weill Medical College of Cornell University, New York, New York, USA

**Keywords:** HIV-1 Env trimer, liposome, nanoparticle presentation, self-assembling protein nanoparticle, virus-like particle

## Abstract

**Purpose of review:**

We will discuss recent advances in the development of nanoparticle vaccines presenting HIV-1 envelope trimer vaccines and the immunological mechanisms by which they act.

**Recent findings:**

The multivalent presentation of Env trimers on nanoparticles is a promising strategy to increase Env immunogenicity. Recent studies have shed light on how Env nanoparticles increase lymph node trafficking and germinal center formation by using the lectin-mediated complement pathway and enhancing the interaction with naïve B cells. Meanwhile, research on different nanoparticle platforms has resulted in improved designs, such as liposomes with improved stability, and the emergence of novel platforms such as protein nanoparticles that self-assemble *in vitro*. Immmunogenicity studies with these nanoparticles delineate the advantages and expose the limitations of the different nanoparticle platforms.

**Summary:**

It is becoming increasingly clear that HIV-1 vaccine research might benefit greatly from using nanoparticles presenting Env trimers, particularly during the priming stage of immunization. Among the different nanoparticles that are being pursued, in vitro-assembling nanoparticles allow for greater control of Env quality making them a promising nanoparticle platform.

## INTRODUCTION

With around 2 million new cases of HIV worldwide the need for a vaccine that can prevent infection remains as high as ever. To deal with the large sequence diversity of HIV-1 it is widely accepted that a successful vaccine should induce broadly neutralizing antibodies (bNAbs) [1,2]. These bNAbs recognize and bind epitopes that are conserved among a wide range of HIV-1 genotypes. The only target for (b)NAbs is the envelope glycoprotein (Env), a trimer of heterodimers, consisting of three gp120 and three gp41 subunits that interact noncovalently. Over the years various forms of recombinantly expressed Env have been used in attempts to generate a vaccine that could induce NAbs. An important breakthrough was the development of native-like trimers exemplified by the prototype BG505 SOSIP.664 trimer [3]. These soluble and stabilized native-like mimics of functional Env present on infectious virus were the first immunogens to elicit potent and consistent NAb responses against neutralization-resistant clinically relevant (Tier-2) viruses in animal models [4–6]. Since then, structure based-design has resulted in the development of native-like Env trimers that have improved stability, increased yield and reduced exposure of nonneutralizing Abs [4,5,7–10]. Despite these improvements, native-like Env trimers generally induce NAbs with narrow specificity that are relatively short-lived. Presenting immunogens in a multivalent way on nanoparticles is a well established strategy to increase their immunogenicity. Here, we review the most recent advances in Env nanoparticle design and the immunological observations made using them. 

**Box 1:**
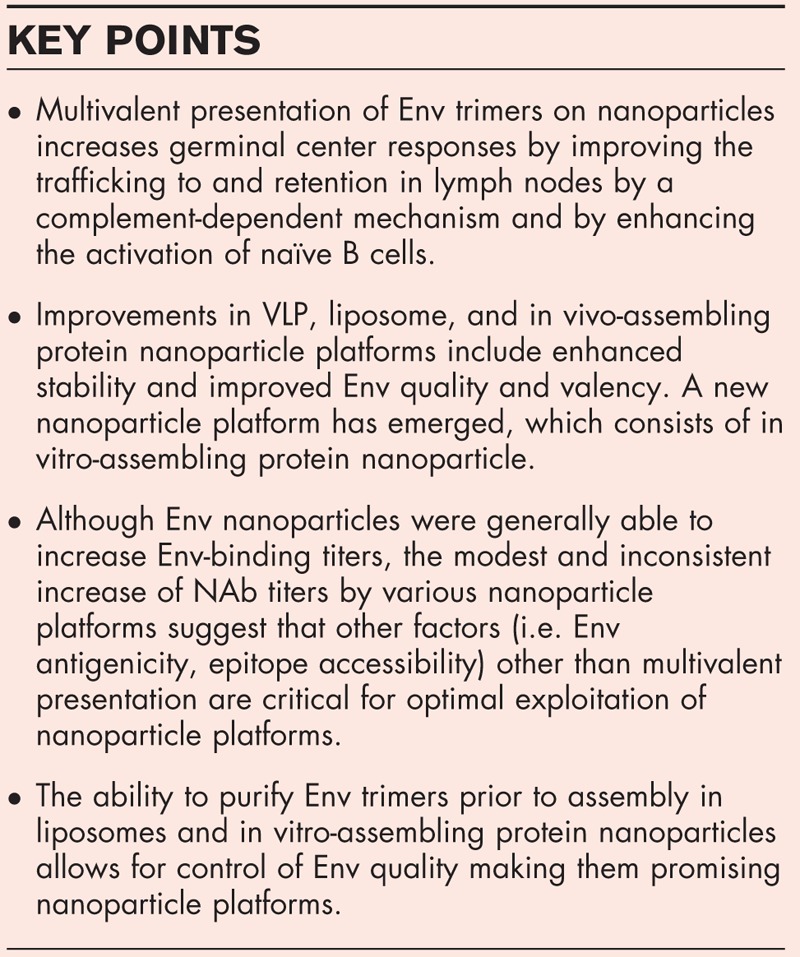
no caption available

## IMMUNOLOGICAL MECHANISMS OF ACTION OF NANOPARTICLES

The success of the commercially available HPV and HBV vaccines and the promising preclinical data for influenza, EBV and malaria nanoparticle vaccines, reinforce the paradigm that multivalent presentation of an immunogen can enhance its immunogenicity [11–14]. In light of this, Env trimers have been presented on various nanoparticle platforms of which several have shown clear improvements in Env immunogenicity. Meanwhile, the immunological mechanisms of action of nanoparticle vaccines are being elucidated and the recent findings are discussed here. The improved immunogenicity of nanoparticle immunogens is rooted in several immunological processes including antigen trafficking to lymph nodes, activation of antigen-specific B cells, and uptake by and activation of antigen presenting cells [15]. It is becoming increasingly clear that the benefit of nanoparticles is a result of the enhancement of multiple immunological processes involving both innate and adaptive responses.

To mount an antibody response, an immunogen has to find its way into the draining lymph nodes where it will interact with B cells and follicular dendritic cells (fDCs). In the case of direct lymphatic drainage, antigens will drain from the administration site by interstitial flow towards a lymphatic vessel. As particulate antigens have lower diffusion rates they will be delivered more effectively than monomeric antigens [16]. Recently, Tokatlian *et al.* provided new insights into the immunological mechanisms behind the improved trafficking to lymph nodes of nanoparticles carrying highly glycosylated antigens such as Env [17^▪▪^]. Nanoparticles presenting Env trimers where present in lymph node follicles, germinal centers, and on fDCs in much higher quantities than their soluble counterparts. This was shown to be largely dependent on complement, as colocalization of particles on fDCs was significantly reduced in mice lacking complement component C3 or its receptors Cr1/2. They further showed that mannose-binding lectin bound significantly better to nanoparticles than soluble immunogens and that the mannose-binding lectin pathway mediated the observed fDC targeting [17^▪▪^]. Thus, as a result of increased avidity by multivalent presentation of Env, nanoparticles more efficiently initiate the mannose-binding lectin – pathway resulting in higher deposition of complement. This subsequently leads to more efficient trafficking to lymph node follicles and presentation on fDCs.

Once in the lymph nodes, nanoparticles will bind more avidly to B-cell receptors (BCRs) on naive cognate B cells than soluble immunogens, enhancing BCR clustering, intracellular BCR-mediated signaling, and subsequent activation of the B cell. Indeed, several studies using Env nanoparticles and Env-specific B cells show that nanoparticle presentation enhances B-cell activation *in vitro* (Brouwer *et al.* in revision) [8,18^▪^,^19^,^20^,^21^]. The ability to more efficiently activate cognate B cells may be of particular relevance in the context of HIV-1 immunogens as bNAb precursors are generally highly infrequent and have low affinity for Env [22]. Recently, Abbott *et al.*[23^▪▪^] tested the effects of immunogen affinity and avidity on germinal center formation in mice that had a range of precursor frequencies of a specific germline B cell. At physiological affinities (0.5 μM K_D_) and frequencies (one in 1 × 10^6^ cells), nanoparticles were able to expand specific germline B cells whereas with the soluble counterpart these B cells were undetectable. Furthermore, even with 10-fold lower precursor frequencies and a 16,000× lower affinity, nanoparticle immunogens outcompeted the monomer with respect to the quantity of detectable specific B cells 8 days post immunization [23^▪▪^].

Inducing antigen-presenting cells to migrate to lymph nodes is a crucial process in the development of a humoral response as they prime cognate follicular T helper cells that drive B-cell affinity maturation in the germinal centers. Nanoparticles have been shown to increase uptake by and activation of dendritic cells over soluble antigen, with a marked dependency on nanoparticle size [24–27]. The improved uptake of particles with a similar size to that of viruses (20–200 nm) has led to speculations that DCs have evolved to recognize virus-sized particles [26,28,29]. However, in the case of receptor-mediated endocytosis the higher avidity by nanoparticle immunogens may also play a role in the enhanced uptake [30]. To what extent and by which mechanism Env nanoparticles increase antigen presentation remains to be studied.

## DIVERSE NANOPARTICLE PLATFORMS FOR HIV-1 ENV TRIMER PRESENTATION

The immunological arguments presented above provide a sound rationale for presenting Env trimers on nanoparticles. Although considerable progress has been made, several problems, including poor *in vivo* stability of Env-nanoparticles, difficulties in ensuring and preserving native-like Env structure, and production issues have limited the improvements in Env immunogenicity conferred by nanoparticle presentation so far. Below, we discuss the main nanoparticle platforms that are currently being explored.

### Virus-like particles

One of the earliest nanoparticles that have been explored are virus-like particles (VLPs). These multiprotein, enveloped particles are produced by cotransfecting the *gag* and *env* gene, resulting in the formation of replication-incompetent particles that mimic the shape and conformation of the HIV-1 virus. However, HIV-1 particles generally only present on average 7–14 Env molecules per particle of which a substantial fraction is nonfunctional with a nonnative structure, including uncleaved and dissociated forms of Env [31–33]. These features, which have been implicated in HIV-1 immune evasion [34,35], suggest that rather than exactly mimicking the natural virus, a successful VLP platform should present more stable and more homogeneous native-like trimers and at higher density than HIV-1 itself.

Crooks *et al.*[36,37] have addressed several of these issues by first stabilizing the Env trimer by addition of a disulfide-bond (SOS) between gp120 and gp41, then removing uncleaved and other nonnative forms of Env from the VLP surface by subjecting them to a cocktail of glycosidases and proteases. The fact that, in contrast to untreated VLPs, the protease-digested VLPs were able to induce some level of heterologous Tier-2 virus neutralization, albeit inconsistently and weakly, suggests the importance of improving the structural homogeneity of expressed trimeric Env on HIV-1 VLPs [37,38]. A common strategy to increase the quantity of Env on the VLP surface is truncating the cytoplasmic tail. Recently, Stano *et al.*[39] were able to further increase Env quantity by generating a VLP production platform that yielded VLPs with more than 100 Env trimers on the surface. How these higher valency VLPs perform *in vivo* remains to be addressed. An interesting approach to improve the immunogenicity of Env VLPs was introduced by Elsayed *et al.*[40], who generated Env VLPs that contained helper T-cell epitopes of tetanus toxoid. Pre-immunization of mice with tetanus toxoid led to significantly increased Env-specific IgG titers, which was shown to be caused by the intrastructural help conferred by the helper T-cell epitope(s) [40]. The elegance of this approach lies in the fact that humans have been vaccinated against tetanus during childhood and that the tetanus memory T cells can be harnessed to help HIV-1 Env immunogenicity, at least in theory.

Despite these recent advances, producing Env VLPs to yields that are sufficient for testing in larger animal models (rabbits, macaques) remains a critical bottleneck. Although the yield of Env-VLPs could be increased by optimizing plasmid combinations, it was estimated that per unit time, more than 100-fold less doses could be produced compared to SOSIP trimers [38]. Combining the described strategies that enhance trimeric integrity and quantity of presented Env and finding ways to increase VLP expression will probably be crucial to solve the multiple limitations Env VLPs have.

### Liposomes

Liposomes are unilamellar or multilamellar vesicles, which consist of a bilayer of (phospho)lipids, cholesterol, and other amphipathic molecules. Various types of liposomes have been used to multivalently present viral antigens, including the FDA-approved influenza (Inflexal V) and hepatitis A (Epaxal) vaccines [41–44]. In the context of HIV-1 Env, liposomes are an attractive nanoparticle platform as they allow purification of native-like Env prior to nanoparticle formation.

Early generations of liposomes used in HIV-1 Env research used lipids bearing Ni^2+^ at the polar head group. Purified Env trimers with a C-terminal His-tag could then be noncovalently coupled to the liposome. This strategy was applied to various Env trimers and showed that the Env-liposomes were superior at B-cell activation than the parental soluble trimers *in vitro*[8,20,45]. Furthermore, when administered to rhesus macaques, Env-liposomes were able to increase the quantities of follicular T helper cells and proliferating B cells in germinal centers, whereas soluble trimers were unable to do so [45]. In addition, Env-liposomes induced modestly higher autologous NAb responses than the parental trimer. However, the noncovalent Env-liposome linkage through Ni^2+^ and His-tag proved instable *in vivo* as these liposomes were unable to preserve the Env trimers on their surface [6,18^▪^,^19^].

To overcome the issue of Env dissociation, next-generation Env-liposomes were developed that contain lipids with a maleimide group allowing for covalent coupling of trimers using a C-terminal cysteine. This strategy markedly increased the stability of Env conjugation. Although Env-liposomes generated using Ni^2+^-His interactions where devoid of trimers after a 4-day incubation in 20% mouse serum, Env-liposomes generated through maleimide-cysteine cross-linking maintained Env on their surface [18^▪^,^19^]. Moreover, the later types of Env-liposomes significantly increased the percentage of germinal center (GC) B cells in the draining lymph nodes of immunized mice and induced significantly higher binding Ab titers than the former liposomes and soluble trimers [19]. However, despite these improvements, rhesus macaques immunized with a stabilized Env trimer conjugated to liposomes by maleimide-cysteine cross-linking developed significantly lower autologous NAbs than the soluble counterpart and the authors speculated that the stability of these liposomes remained inadequate [6]. Env retention on liposomes could be further enhanced by increasing the maleimide concentration from 5 to 15%, introducing sphingomyelin and exchanging the lipid DSPC to DPPC. Although an initial immunization study in mice showed that the increased stabilization further enhanced the percentage of GC B cells, it remains to be tested whether these liposomes are able to increase NAb responses in a more relevant animal model [18^▪^].

### In vivo-assembling protein nanoparticles

The discovery and design of proteins that self-assemble into well ordered complexes with three-fold symmetry has opened the door for the development of protein-only nanoparticles presenting Env trimers [46]. In recent years, the genetic fusion of Env to the termini of these one-component self-assembling proteins has yielded various recombinantly expressed nanoparticles that present 8 or 20 trimers [12,13,21,47,48,49^▪^]. The well ordered geometry of the presented Env and the ease of production, which can be scaled up using current translational methods, make these nanoparticles an attractive platform.

One of the first self-assembling proteins to be used in combination with Env is ferritin. Genetic fusion of Env to ferritin yields a 24 subunit nanoparticle of around 30 nm presenting eight trimers [47]. Two subsequent studies by He *et al.* and Sliepen *et al*. showed that Env trimers presented on ferritin nanoparticles induced significantly higher binding antibody and autologous NAb responses than single trimers in rabbits (Sliepen *et al*. in press) [49^▪^]. The benefit of the nanoparticles was evident particularly after the priming immunization (Sliepen *et al*. in press).

As a higher number of Env trimers per particle may further improve the immunogenicity of protein nanoparticles, several one-component nanoparticles have been described that present 20 Env trimers. Fusion of Env to E2p, a 60-subunit particle from *B. Stearothermophilus*, yielded particles that, in contrast to ferritin nanoparticles, were able to bivalently interact with certain bNAbs. However, the yields of these particles was low and Env antigenicity was not optimal [49^▪^]. The computationally designed I3–01 nanoparticle platform has also been used to present 20 Env trimers [49^▪^,^50^]. T-cell epitopes to further enhance Env immunogenicity were also included in these nanoparticles. Although soluble Env failed to induce autologous neutralization in mice, Env presented on I3–01 with a T-cell epitope induced modest autologous NAb responses in two of eight rabbits.

**FIGURE 1:**
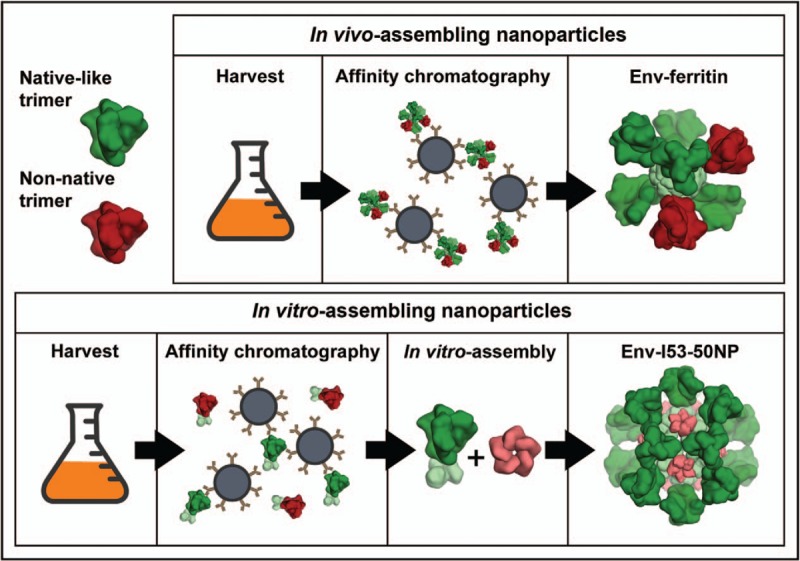
In vitro*-*assembling (two-component) nanoparticles allow for greater control over Env quality than in vivo*-*assembling (one-component) nanoparticles. In contrast to in vivo-assembling nanoparticles such as Env-ferritin (top panel) or VLPs presenting Env, in vitro-assembling nanoparticles such as Env-liposomes or Env-I53-50 nanoparticles (bottom) allow purification of Env trimers prior to assembly ensuring that native-like trimers (green) are selected and nonnative trimers (red) are excluded from the nanoparticles.

Although several in vivo-assembling protein nanoparticles were able to augment Env immunogenicity, the improvements were modest and inconsistent, at best. One issue with ferritin nanoparticles (and presumably also E2p and I3-01 nanoparticles), and one that might be inherent to most *in vivo*-assembling platforms, is that once the nanoparticles are assembled, presumably in the endoplasmic reticulum, furin has no easy access to its cleavage site between gp120 and gp41, resulting in incomplete precursor cleavage (Sliepen *et al*. in press) [47,51]. This combined with the inability to exclude nonnative Env trimers from the nanoparticles (Fig. 1), may lead to the exposure of highly immunodominant epitopes that are irrelevant to neutralization of primary, neutralization-resistant (Tier 2) viruses, and might even distract from NAb epitopes [52]. Indeed, Env-ferritin nanoparticles usually augment NAb responses against lab-adapted and highly neutralization sensitive viruses (Tier 1) more than NAb responses against primary Tier 2 viruses, consistent with the concerns described above (Sliepen *et al*. in press; Brouwer *et al.* in revision) [47,51].

### In vitro-assembling protein nanoparticles

In vitro-assembling nanoparticle systems offer advantages as they allow more control over the quality of the Env trimers by allowing the purification of native-like trimers prior to assembly. The recent development of protein nanoparticles that self-assemble *in vitro* has opened up new platform for nanoparticle vaccines [53,54]. Bale *et al.*[53] used computational protein structure prediction to design a large number of icosahedral particles which assembled highly-efficiently *in vitro*. One nanoparticle design in particular, designated I53-50 and consisting of 20 trimeric (I53-50A) and 12 pentameric (I53-50B) subunits, was amenable for fusion to viral glycoproteins [55^▪▪^]. We fused Env trimers to the trimeric I53-50A component, and subsequent mixing with the pentameric I53-50B component produced monodisperse and well ordered nanoparticles presenting twenty trimers (Env-I53-50NP). The assembly efficiency and Env quality were excellent (Brouwer *et al.* in revision).

Immunization studies in rabbits showed that Env-I53-50NPs significantly increased Env immunogenicity, including NAb responses (Brouwer *et al.* in revision). This was particularly evident after the priming immunizations, when the autologous NAb responses were 40-fold higher in the Env-I53-50NP group compared to the single trimer group. Furthermore, Env-I53-50NPs were superior over Env-ferritin particles in augmenting autologous NAb responses, while suppressing responses against nonneutralizing epitopes, confirming the quality of the presented Env trimers.

However, we found that the benefit of nanoparticle presentation effect was dependent on the location of the immunodominant epitope of the particular Env used. When Env-I53-50NPs with an immunodominant epitope proximal to the trimer base were used, there was no beneficial effect of nanoparticle presentation (Brouwer *et al.* in revision). This proved to be a result of poor epitope accessibility and showed that epitope location and accessibility are parameters that should be considered in nanoparticle vaccine design.

## CONCLUSION

Recent years have not only seen the emergence of new Env-nanoparticle platforms but also increased the understanding in the way nanoparticles enhance immunogenicity. These recent immunological insights as well as outcomes from immunization studies indicate that nanoparticles will be particularly beneficial during the priming immunization when the low affinity interactions of naïve B cells with immunogens benefit from enhanced avidity (Brouwer *et al.* in revision; Sliepen *et al*. in press) [18^▪^,^23^^▪▪^]. Within the competitive environment of the germinal center, it might also be critical to optimize presentation of bNAb epitopes and eliminate non-NAb epitopes. Therefore, the ability to purify native-like trimers prior to nanoparticle formation might be a critical advantage of liposomes and two-component self-assembling nanoparticles and make these particularly attractive platforms moving forward.

## Acknowledgements

*We acknowledge Neil King for kindly sharing the Pymol script that was used to the create the Env trimer and nanoparticle structures in*Fig. 1.

### Financial support and sponsorship

*Work by the authors in this area is supported by the U.S. National Institutes of Health Grant P01 AI110657; by the Bill and Melinda Gates Foundation through the Collaboration for AIDS Vaccine Discovery (CAVD), grants OPP1111923 and OPP1132237; by the European Union's Horizon 2020 research and innovation programme under grant agreement No. 681137; and by a Vici grant from the Netherlands Organization for Scientific Research (NWO)*.

### Conflicts of interest

There are no conflicts of interest.

## REFERENCES AND RECOMMENDED READING

Papers of particular interest, published within the annual period of review, have been highlighted as:

▪ of special interest
▪▪ of outstanding interest
